# Purification of Derivatized Oligosaccharides by Solid Phase Extraction for Glycomic Analysis

**DOI:** 10.1371/journal.pone.0094232

**Published:** 2014-04-04

**Authors:** Qiwei Zhang, Henghui Li, Xiaojun Feng, Bi-Feng Liu, Xin Liu

**Affiliations:** Britton Chance Center for Biomedical Photonics at Wuhan National Laboratory for Optoelectronics–Hubei Bioinformatics and Molecular Imaging Key Laboratory, Systems Biology Theme, Department of Biomedical Engineering, College of Life Science and Technology, Huazhong University of Science and Technology, Wuhan, China; University of Patras, Greece

## Abstract

Profiling of glycans released from proteins is very complex and important. To enhance the detection sensitivity, chemical derivatization is required for the analysis of carbohydrates. Due to the interference of excess reagents, a simple and reliable purification method is usually necessary for the derivatized oligosaccharides. Various SPE based methods have been applied for the clean-up process. To demonstrate the differences among these methods, seven types of self-packed SPE cartridges were systematically compared in this study. The optimized conditions were determined for each type of cartridge and it was found that microcrystalline cellulose was the most appropriate SPE material for the purification of derivatized oligosaccharide. Normal phase HPLC analysis of the derivatized maltoheptaose was realized with a detection limit of 0.12 pmol (S N^−1^ = 3) and a recovery over 70%. With the optimized SPE method, relative quantification analysis of *N*-glycans from model glycoproteins were carried out accurately and over 40 *N*-glycans from human serum samples were determined regardless of the isomers. Due to the high stability and sensitivity, microcrystalline cellulose cartridge showed potential applications in glycomics analysis.

## Introduction

Glycosylation represents one of the most complex and widespread post-translational modifications of human proteins. *N*-linked and *O*-linked glycosylations are two major patterns that are related to the structure and function of the glycoproteins [1]–[3]. Aberrant glycosylation profile may lead to dramatic changes of the proteins in the activity, distribution as well as stability [4]–[7]. Thus, the analysis of glycosylation is extensively conducted in biological and clinical research as well as in pharmaceutical industry [8]–[10]. Different methods have been reported, including high performance liquid chromatography (HPLC) [11], [12], capillary electrophoresis [13], [14] and high pH anion-exchange chromatography [15], [16] with electrochemical, optical or mass spectrometric (MS) detection [17], [18]. To enhance the detection sensitivity, oligosaccharides are usually derivatized prior to analysis. Most of such derivatization reactions can be accomplished by coupling the reagents with the reducing end of saccharides [17], [19]. During this step, reagents, including salts, derivatization reagents and solvent are normally presented in large excess. Thus, a clean-up procedure has to be implemented to remove the excess reagents that often interfere with the following detection [20].

Various methods have been applied for the clean-up processes, such as paper chromatography, gel filtration, precipitation and solid-phase extraction (SPE) [21]–[24]. Among these strategies, SPE based approaches have been widely employed due to its diverse stationary phases, rapid and simple operation, and high throughput [25]. There are two major types of stationary phases for SPE methods: carbon and hydrophilic materials. As a typical carbon material, porous graphitized carbon (PGC) shows good performance in the purification of non-derivatized glycans [8], [26]. However, it presents some drawbacks for labeled samples. The elution for PGC required the use of solutions containing 0.1% trifluoroacetic acid (TFA), which could cause the loss of sialic acid residues if it was not removed properly [27]. Moreover, most of the labels consist of aromatic nucleus, which have strong adsorption to PGC, resulting in difficulty in the removal of excess labels from the derivatized oligosaccharides. In contrast, hydrophilic interaction chromatography (HILIC) SPE method can overcome this problem. In this method, the glycans are retained on stationary phases by hydrophilic interaction, whereas excess labels can be removed due to their lower hydrophilicity than the glycans [20]. Therefore, HILIC SPE method is becoming the most suitable strategy for the purification of derivatized glycans. Several stationary phases have been reported for this method, including DPA-6S, microcrystalline cellulose (MCC) and cotton [27]–[29]. Discovery DPA-6S column showed good performance in the recovery of labeled oligosaccharides [28]. The MCC-based SPE method showed high reproducibility for the fast sample purification [27]. But MCC was always used in the loose state without being compressed, which might reduce sample recovery. Cotton SPE microtips could remove salts, most nonglycosylated peptides, and detergents such as sodium dodecyl sulfate from microscale samples [29]. Although these HILIC SPE methods were widely employed for the purification of derivatized oligosaccharides, a complete comparison of these methods on terms of recovery and reproducibility has not yet been reported before. In this study, we systematically compared the performances of different materials as the stationary phases of SPE cartridges, including DPA-6S, MCC, DSC-CN, DSC-Si, DSC-NH_2_, DSC-Diol and ZIC-HILIC. Since it is difficult to fill cotton into the cartridge evenly, we exclude the test of cotton. The aim of this study is to find out the most appropriate HILIC SPE method and to establish a standard strategy for the purification of labeled oligosaccharides.

By comparing the recovery and reproducibility, it was found that MCC was the most appropriate material and chosen for the next experiments. The washing conditions were optimized and the differences of two reaction conditions were demonstrated by a relative quantitative research. The MCC cartridge was further applied to the purification of biological samples followed by HPLC analysis. Results showed that MCC held high potential for the analysis of complex and trace-level samples such as the human serum.

## Materials and Methods

### Chemicals and Reagents

DSC-Si, DSC-CN, DSC-NH_2_, DSC-Diol, ZIC-HILIC, DPA-6S, maltoheptaose, 2-aminobenzoic acid (2-AA), Fetuin, ribonuclease B (RNase B) and human serum were purchased from Sigma-Aldrich (MO, U.S.A.). *N*-glycosidase F (PNGase F) and endoglycosidase buffer pack (EBP) were obtained from New England Biolabs (MA, U.S.A.). Deionized water was purified using a Milli-Q device (Millipore, MA, U.S.A.). Acetic acid, formic acid (FA), TFA, dimethyl sulfoxide (DMSO), sodium cyanoborohydride, ammonium formate, sodium acetate, boric acid, methanol, acetone, hexane, ethanol, acetonitrile (ACN) and MCC were from Sinopharm Chemical Reagent Co. Ltd (Shanghai, China). HPLC grade ACN was from Merck KGaA (Darmstadt, Germany). Empty SPE cartridges of 1.0 mL were purchased from Biocomma (Guangdong, China). The human serum samples, including 6 lung cancer patients and 6 healthy controls, were donated by Tongji Hospital (Tongji Medical College Huazhong University of Science and Technology). The study was carried out in accordance with the Helsinki Declaration. Informed consent was obtained from the participants in accordance with the study protocols approved by the Ethics Committee of Huazhong University of Science and Technology. All subjects had provided their written informed consent to participate in this study.

### Preparation of *N*-glycans

*N*-glycans were prepared from model glycoproteins and human serum samples with PNGase F as reported previously with slight modifications [30]. Glycoproteins of 10 μL dissolved in deionized water or serum samples were mixed with 90 μL of 20 mM sodium phosphate (pH = 7.5) that contained 0.13% dodecyl sulfate sodium salt and 10 mM dithiothreitol from EBP. The proteins were denatured at 100°C for 10 min and then cooled to the room temperature, which were further mixed with 12 μL of 10% octylphenoxypolyethoxyethanol (NP-40) from EBP. PNGase F stock solution of 5 μL was added to the solution and incubated at 37°C for 16 h. The reaction mixture was then dried in a vacuum concentrator (Eppendorf, Germany) and stored at −20°C before derivatization.

### Derivatization with 2-aminobenzoic acid

The fluorescent label 2-AA was widely used for HPLC analysis of glycans. Two typical reaction conditions were used in this study [15], [19]. In condition 1, 20 μL of solution, consisting of 0.35 M 2-AA and 1.0 M sodium cyanoborohydride in 3/7 (v/v) acetic acid/DMSO, was added to the dry sample. The mixture was incubated at 65°C for 2.5 h, and the acetic acid was then removed in the vacuum concentrator to stop the reaction. In condition 2, 1.0 mL of solution, consisting of 0.22 M 2-AA, 0.32 M sodium cyanoborohydride, 4% sodium acetate (w/v) and 2% boric acid (w/v) in methanol, was mixed with 30 μL of sample dissolved in water. The mixture was incubated at 80°C for 45 min, and the solvent was then removed in the vacuum concentrator. The derivatized oligosaccharides were refrigerated at −20°C for storage.

### The self-packed SPE cartridges

Seven kinds of materials were used to fill the SPE cartridges, including DPA-6S, MCC, DSC-Si, DSC-Diol, DSC-CN, DSC-NH_2_ and ZIC-HILIC. All materials were packed in the SPE cartridges using the same method. For example, 100 mg DPA-6S suspended in deionized water was filled into 1.0 mL empty SPE cartridge with a frit at the bottom. The cartridge was dried at 60°C for 4 h. After cooling to the room temperature, another frit was plugged into the cartridge.

### The purification of 2-AA derivatized oligosaccharides

The 2-AA derivatized maltoheptaose was diluted by 90 μL of 80% (v/v) ACN and purified using SPE cartridge. Different cartridges were washed with 3.0 mL water and 3.0 mL ACN, followed by 3.0 mL of the corresponding solution in Table 1. Each sample mixture was loaded on a cartridge and then washed with the corresponding solution. The eluted fractions were collected, dried in the vacuum concentrator and stored at −20°C before further analysis. The *N*-glycans released from model glycoproteins and human serum samples were purified by MCC cartridge with the optimized purification condition.

**Table 1:** The purification conditions for each type of SPE cartridge.

Cartridge	Washing solution	Elution solution
DPA-6S	95% ACN (v/v), 5.0 mL	20% ACN (v/v), 1.0 mL
MCC	80% ACN and 3% FA (v/v), 5.0 mL	Water, 1.0 mL
DSC-CN	95% ACN (v/v), 10.0 mL	Water, 1.0 mL
DSC-Si	Acetone/Ethanol = 1/1 (v/v), 15.0 mL	Water, 1.0 mL
DSC-NH_2_	80% ACN (v/v), 15.0 mL	5% FA (v/v), 1.0 mL
DSC-Diol	Hexane/Acetic acid = 3/2 (v/v), 8.0 mL	20% ACN (v/v), 1.0 mL
ZIC-HILIC	80% ACN and 5% FA (v/v), 10.0 mL	Water, 1.0 mL

### Normal phase HPLC analysis

The derivatized oligosaccharide samples were dissolved in 100 μL of solution consisting of ACN/100 mM HCOONH_4_ (1∶4, v/v). 20 μL of the above solution was analyzed by a Prominence LC-20A (Shimadzu, Japan). Normal phase HPLC profiling of 2-AA derivatized oligosaccharides was performed with Cosmosil 5NH_2_-MS column (5.0 μm, 4.6 mm I.D.×250 mm L., Nacalai Tesque, Japan). A binary gradient was applied using 100 mM HCOONH_4_ (pH = 4.4) as solvent A and ACN/solvent A (4∶1, v/v) as solvent B. The following gradient conditions were mainly applied for *N*-glycans released from biological samples: 0 min 70% solvent B; 5 min 70% solvent B; 180 min 5% solvent B; 185 min 5% solvent B; 186 min 70% solvent B. The fractions from the human serum sample were collected and dried in the vacuum concentrator. The column temperature was 35°C (CTO-20A column oven, Shimadzu, Japan) and the flow rate was maintained at 1.0 mL min^−1^. The separation results were monitored using a fluorescence detector (RF-10AXL, Shimadzu, Japan) with 360 nm excitation and 419 nm emission [19].

### NanoLC-MS analysis

The HPLC fractions were dissolved in 40 μL of 5% ACN solution containing 0.1% FA (v/v). Each 2 μL of solution was analyzed using a nanoflow liquid chromatography mass spectrometry (nanoLC-MS) system which consisted of a NanoLC Ultra System (Eksigent, U.S.A.) and a TripleTOF 5600 System (AB SCIEX, U.S.A.). Fractions were loaded onto a ChromXP nanoLC Trap column (350 μm I.D.×5 mm L.; C18, 3 μm, 120 Å; Eksigent, U.S.A.) with 5% ACN solution containing 0.1% FA (v/v) as solvent A for 10 min at a flow rate of 2.0 μL min^−1^, which were then injected into the ChromXP nanoLC column (75 μm I.D.×150 mm L., C18, 3 μm, 120 Å, Eksigent, U.S.A.) at a flow rate of 300 nL min^−1^ before the samples were detected by electrospray ionization mass spectrometry (ESI-MS). A gradient program was employed for the nanoLC separation, starting at 5% solvent B consisting of 95% ACN solution and 0.1% FA (v/v), which constantly rose to 50% over 15 min. The column was then washed with 80% solvent B for 5 min and 5% solvent B for 5 min prior to next sample. The solvent was evaporated at 150°C with a nitrogen stream of 3 L min^−1^ and the ion spray voltage floating was set to 2300 V. MS was operated in positive-ion mode with mass range of 500–2000 m/z. MS/MS was acquired in an automated data-dependent acquisition mode with charge numbers from 2 to 5 and mass range of 100–2000 m/z. The ESI-MS data was analyzed using PeakView 1.2 software (AB SCIEX, U.S.A.).

## Results and Discussion

### Optimized purification conditions for each type of SPE cartridge

2-AA derivatized maltoheptaose of 0.15 μg was purified using seven types of SPE cartridges. The purification conditions for each stationary phase were compared and optimized in this study (Table S1 and Figure S1 in File S1). As a result, the washing solution for DPA-6S could not contain water more than 5% (v/v). If the volume percentage of water went up to 10%, the recovery of derivatized oligosaccharides would decline significantly. For MCC, literature has shown that a small amount of acid in the washing solution could enhance the efficiency in the enrichment of glycopeptides [31]. Similarly, acid played an important role in the purification of glycans. The recovery of maltoheptaose increased with increasing concentration of acid. However, excess acid might improve the hydrophilicity of some impurities, leading to increased amount of impurities in the eluted fractions. Comparison study of the washing solutions containing FA and TFA was then conducted to determine the optimal type and concentration of the acid. Results showed that FA was better than TFA (data not shown) and the solution containing 3% (v/v) FA yielded about 10% increase in the recovery relative to the solution without acid. In addition to DPA-6S and MCC cartridges, another potential material was DSC-CN, for which the optimized condition was similar to DPA-6S. For DSC-NH_2_ cartridge, the elution solution must contain acid (e.g. 5.0% FA (v/v)) so that it could reach the capability of the DSC-Si column in terms of average recovery. Otherwise, maltoheptaose could not be eluted from the DSC-NH_2_ column. In comparison to above materials, DSC-Diol and ZIC-HILIC columns yielded the lowest recoveries in our study.

**Figure 1:**
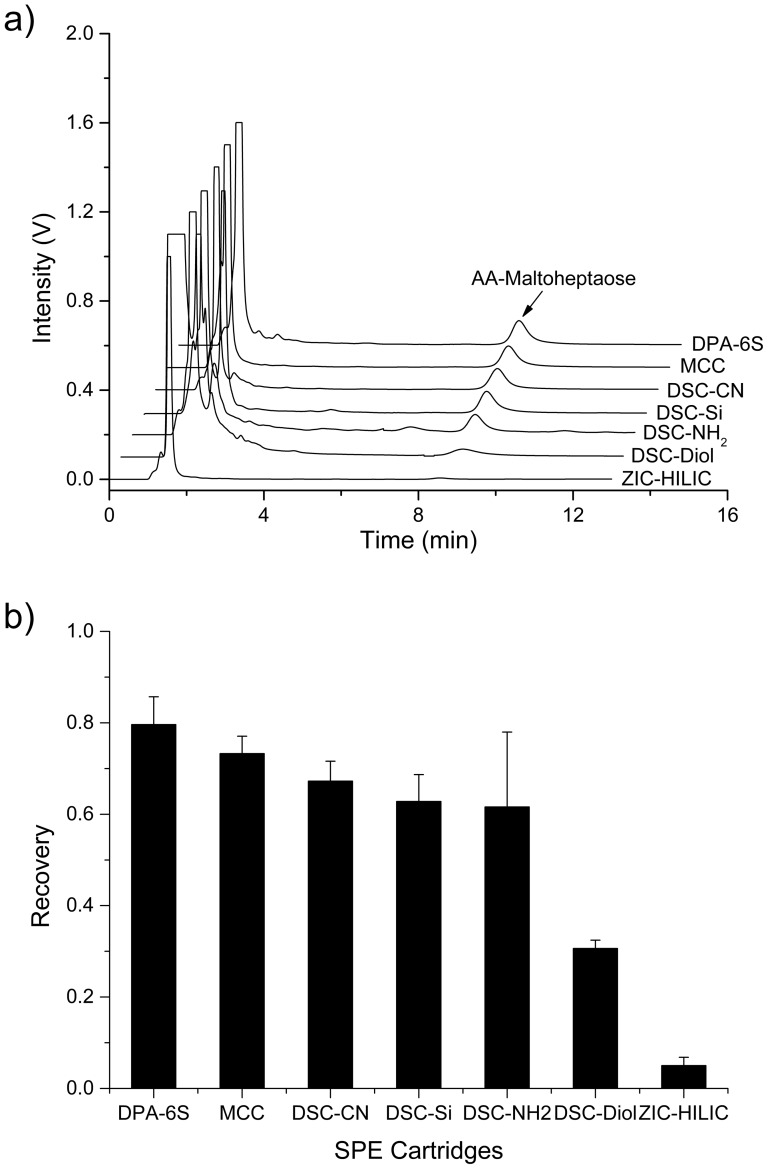
Purification performance of the seven stationary phases under the optimized conditions shown in Table 1. (a) The HPLC spectra of 2-AA derivatized maltoheptaose (0.15 μg) for each type of SPE cartridge. (**b**) The recovery and RSD for each type of SPE cartridge, showed the different purification effect (n = 3).

The optimized purification conditions were listed in Table 1 and Figure 1a showed the HPLC spectra, corresponding to Table 1. The recoveries and relative standard deviations (RSD) of all types of cartridges were given in Figure 1b with the close removal efficiency of impurities. As shown in Figure 1b, the recoveries of DPA-6S and MCC were 79.6% and 73.3%, which were the highest among the seven materials. DSC-Diol and ZIC-HILIC produced the lowest recoveries, which were 30.6% and 5.0%. DSC-CN, DSC-Si and DSC-NH_2_ yielded recoveries of 67.3%, 62.8% and 61.6%, respectively. Furthermore, DSC-NH_2_ column was very unstable, showing the highest RSD of more than 15%, while all other types of cartridges gave a RSD of approximately 6% or lower. Thus, by comparing the recoveries and the reproducibilities of the seven types of cartridges, it was found that DPA-6S and MCC appeared to be more superior in the purification of derivatized oligosaccharides.

### The evaluation of self-packed MCC cartridge

Cotton could remove salts, most nonglycosylated peptides, and detergents from released *N*-glycan samples [29]. Since MCC has similar properties to cotton, it would be more suitable than DPA-6S for the clean-up of biological samples that were directly derived after digestion. A previously reported labeling method was used in this study after digestion of glycoproteins [27], [32]. Labeling reagent was directly added to the released *N*-glycan samples that contained detergent, buffer and PNGase F. In addition to the above advantage, MCC is also easy to obtain and handle. Therefore, MCC was chosen as the optimal stationary phase for the subsequent experiments.

To achieve a good purification effect, some preparations were needed for MCC. After MCC was packed into empty cartridges, it must be washed with deionized water for several times to remove hydrophilic impurities, such as polysaccharides adsorbed on MCC. The cartridge was then dried and stored at room temperature. Just before use, it needed to be compressed to about 8.5 mm (I.D. 5.5 mm), which was the most reasonable height according to our experience. A column higher than 8.5 mm could reduce sample recovery and a column lower than 8.5 mm could lead to slow flow velocity. After preparation of the MCC cartridge, sample mixtures were purified before HPLC analysis. Normal phase HPLC analysis was performed to produce the standard curve. It showed satisfactory linear relationship (R^2^ = 0.9996, n = 3) within a large range and a detection limit of 0.14 ng (c.a. 0.12 pmol, S N^−1^ = 3). The RSD was approximately 5% for repeated experiments and the recovery exceeded 70%. It was proven that our method showed good stability and sensitivity.

### Analysis of *N*-glycans released from glycoproteins

Maltoheptaose of 0.1, 1.0 and 10.0 μg were labeled with 2-AA using both condition 1 and 2. After HPLC analysis, the yield of the derivatization was found approximately the same under both conditions for the three different amounts of maltoheptaose. However, the reaction stability was different for the derivatization of *N*-glycans released from glycoproteins under the two conditions. Model glycoprotein, RNase B of 0.05, 0.13 and 0.20 mg were digested by PNGase F, and the high-mannose type glycans were then directly labeled with 2-AA without purification. The relative abundances of Mannose_5_ *N*-acetylhexosamine_2_ were 50.2%, 49.1% and 49.8% under condition 1, and 49.0%, 46.5% and 44.8% under condition 2 for the three different amounts of RNase B. The relative abundance was maintained constant under condition 1, while it seemed that the relative abundance declined with increasing amount of RNase B under condition 2. Our data have shown different derivatization results for maltoheptaose and oligosaccharides released glycoproteins under condition 2. It was possible that the direct labeling after digestion resulted in the derivatization discrimination. In contrast, condition 1 did not show such derivatization discrimination, which was more suitable for the labeling of mixture constituted of various oligosaccharides with different concentrations. To ensure the reaction stability, condition 1 was applied for the reductive amination of saccharides from released *N*-glycan samples.

The 2-AA derivatized *N*-glycans were analyzed by HPLC with a Cosmosil 5NH_2_-MS column as the stationary phase. High-mannose type oligosaccharides from RNase B and complex type oligosaccharides from Fetuin were separated completely according to different number of residues. For high-mannose type oligosaccharides, the retention time changed with increasing number of mannose residues and rendered a linear relationship (R^2^ = 0.9960). A linear relationship was observed between the retention time and the mass of the 2-AA derivatized oligosaccharides, which was consistent with those previously reported for the permethylated *N*-linked glycans [33]. The relative abundances of oligosaccharides were calculated as 49.7±0.4%, 31.7±0.3%, 7.7±0.3%, 8.3±0.3% and 2.6±0.1% for Mannose_5_ *N*-acetylhexosamine_2_ to Mannose_9_ *N*-acetylhexosamine_2_, respectively. The relative quantity of the five oligosaccharides was slightly different from those reported in other studies, which might result from different sources of RNase B [34]. With the direct labeling method and MCC cartridge as the purification tool, the relative quantitative analysis of *N*-glycans could be accomplished with high stability. For complex type oligosaccharides, they were mainly separated based on the number and linkage of sialic acid residues. Based on the retention time, these glycans were regularly divided into three groups, i.e., mono-, bi- and tri-/tetra sialic acid residues. It seemed that the number of acid residues significantly affected the retention time, thus it was found that the 5NH_2_-MS column could be very suitable for the analytical separation of sialylated glycans due to the feature.

### Profiling of *N*-glycans released from human serum

**Figure 2:**
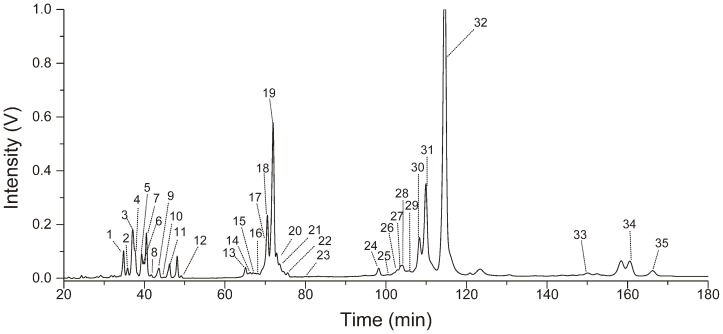
The *N*-glycans released from 10 μL of human serum were separated by HPLC. The resulting 35 peaks were divided into Group 1 (Peak 1–12), Group 2 (Peak 13–23), Group 3 (Peak 24–32) and Group 4 (Peak 33–35).

The chromatogram of 2-AA derivatized *N*-glycans from human serum was shown in Figure 2. The resulting 35 peaks were divided into four groups based on the number of sialic acid residues. Group 1–4 consisted of non-sialylated (Peak 1–12), mono- (Peak 13∼23), bi- (Peak 24∼32) and tri- (Peak 33∼35) sialic acid residues oligosaccharides, respectively. Among these groups, there were about 15-minute intervals in which no peaks were observed. The *N*-glycan compositions and structures of each peak were shown in Table 2. For non-sialylated oligosaccharides, the retention time increased linearly for a specific subclass (e.g. Hex_3_HexNAc_5_Fuc_1_, Hex_4_HexNAc_5_Fuc_1_, Hex_5_HexNAc_5_Fuc_1_, Hex_6_HexNAc_5_Fuc_1_, R^2^ = 0.9992) and were mainly affected by the number of antennas and monosaccharides, particularly, the terminal hexoses residues whose effect seemed to be weak for sialylated oligosaccharides. In addition, fucose residues also affected the retention time. The core fucose residue led to shorter retention time, and the terminal fucose residue would greatly extend the retention time. Some isomers of oligosaccharides, which resulted from the linkage of sialic acid residues, could be determined from Group 2–4 (e.g. Hex_4_HexNAc_5_Sia_1_Fuc_1_, Hex_5_HexNAc_4_Sia_2_Fuc_1_, and Hex_6_HexNAc_5_Sia_3_). Most of peaks corresponded one or two sugars expect for Peak 4, 13, 14 and 19. Moreover, the oligosaccharides from Peak 14 and part of glycans from Peak 19 were isomers. The similarity of component from Peak 13, 14 and 19 suggested that the oligosaccharides with two antennas and a sialic acid residues were easy to form isomers and difficult to be separated under this condition.

**Table 2:** Identification and annotation of 35 peaks shown in Figure 2.

Peak No.	Measured Mass [M+nH]^n+^	Theoretical Mass [M+nH]^n+^	*N*-Glycan Composition
1	691.2651 (n = 2)	691.2662 (n = 2)	Hex_3_HexNAc_3_Fuc_1_
	792.8040 (n = 2)	792.8059 (n = 2)	Hex_3_HexNAc_4_Fuc_1_
2	821.3186 (n = 2)	821.3166 (n = 2)	Hex_3_HexNAc_5_
	894.3469 (n = 2)	894.3456 (n = 2)	Hex_3_HexNAc_5_Fuc_1_
3	772.2952 (n = 2)	772.2926 (n = 2)	Hex_4_HexNAc_3_Fuc_1_
	873.8337 (n = 2)	873.8323 (n = 2)	Hex_4_HexNAc_4_Fuc_1_
4	678.7505 (n = 2)	678.7504 (n = 2)	Hex_5_HexNAc_2_
	902.3449 (n = 2)	902.3430 (n = 2)	Hex_4_HexNAc_5_
	975.3748 (n = 2)	975.3720 (n = 2)	Hex_4_HexNAc_5_Fuc_1_
5	954.8617 (n = 2)	954.8587 (n = 2)	Hex_5_HexNAc_4_Fuc_1_
6	983.3725 (n = 2)	983.3694 (n = 2)	Hex_5_HexNAc_5_
	1056.4034 (n = 2)	1056.3984 (n = 2)	Hex_5_HexNAc_5_Fuc_1_
7	759.7780 (n = 2)	759.7768 (n = 2)	Hex_6_HexNAc_2_
8	758.6212 (n = 3)	758.6190 (n = 3)	Hex_6_HexNAc_5_Fuc_1_
9	753.3058 (n = 3)	753.2873 (n = 3)	Hex_5_HexNAc_5_Fuc_2_
	840.8122 (n = 2)	840.8032 (n = 2)	Hex_7_HexNAc_2_
10	800.7802 (n = 2)	800.8033 (n = 2)	Hex_4_HexNAc_4_
11	921.8337 (n = 2)	921.8296 (n = 2)	Hex_8_HexNAc_2_
12	1002.8583 (n = 2)	1002.8560 (n = 2)	Hex_9_HexNAc_2_
13	747.6234 (n = 3)	747.6155 (n = 3)	Hex_4_HexNAc_5_Sia_1_Fuc_1_
	917.8509 (n = 2)	917.8403 (n = 2)	Hex_4_HexNAc_3_Sia_1_Fuc_1_
	1019.3934 (n = 2)	1019.3800 (n = 2)	Hex_4_HexNAc_4_Sia_1_Fuc_1_
14	801.6339 (n = 3)	801.6332 (n = 3)	Hex_5_HexNAc_5_Sia_1_Fuc_1_
	844.8133 (n = 2)	844.8114 (n = 2)	Hex_4_HexNAc_3_Sia_1_
	946.3534 (n = 2)	946.3510 (n = 2)	Hex_4_HexNAc_4_Sia_1_
	1100.4110 (n = 2)	1100.4064 (n = 2)	Hex_5_HexNAc_4_Sia_1_Fuc_1_
15	855.6534 (n = 3)	855.6508 (n = 3)	Hex_6_HexNAc_5_Sia_1_Fuc_1_
	1027.3801 (n = 2)	1027.3775 (n = 2)	Hex_5_HexNAc_4_Sia_1_
16	807.3073 (n = 3)	807.3049 (n = 3)	Hex_6_HexNAc_5_Fuc_2_
	925.8455 (n = 2)	925.8378 (n = 2)	Hex_5_HexNAc_3_Sia_1_
17	747.6205 (n = 3)	747.6155 (n = 3)	Hex_4_HexNAc_5_Sia_1_Fuc_1_
18	806.9751 (n = 3)	806.9648 (n = 3)	Hex_6_HexNAc_5_Sia_1_
19	801.6332 (n = 3)	801.6332 (n = 3)	Hex_5_HexNAc_5_Sia_1_Fuc_1_
	844.8109 (n = 2)	844.8114 (n = 2)	Hex_4_HexNAc_3_Sia_1_
	855.6551 (n = 3)	855.6508 (n = 2)	Hex_6_HexNAc_5_Sia_1_Fuc_1_
	881.8338 (n = 2)	881.8297 (n = 2)	Hex_5_HexNAc_4_
	946.3511 (n = 2)	946.3510 (n = 2)	Hex_4_HexNAc_4_Sia_1_
	954.8583 (n = 2)	954.8587 (n = 2)	Hex_5_HexNAc_4_Fuc_1_
	1019.3812 (n = 2)	1019.3800 (n = 2)	Hex_4_HexNAc_4_Sia_1_Fuc_1_
	1027.3788 (n = 2)	1027.3775 (n = 2)	Hex_5_HexNAc_4_Sia_1_
	1100.4092 (n = 2)	1100.4064 (n = 2)	Hex_5_HexNAc_4_Sia_1_Fuc_1_
20	1047.8943 (n = 2)	1047.8907 (n = 2)	Hex_4_HexNAc_5_Sia_1_
21	752.9492 (n = 3)	752.9472 (n = 3)	Hex_5_HexNAc_5_Sia_1_
22	1006.8728 (n = 2)	1006.8642 (n = 2)	Hex_6_HexNAc_3_Sia_1_
23	777.6297 (n = 3)	777.6261 (n = 3)	Hex_6_HexNAc_6_
24	830.9769 (n = 3)	830.9718 (n = 3)	Hex_5_HexNAc_4_Sia_2_Fuc_1_
25	782.2915 (n = 3)	782.2859 (n = 3)	Hex_5_HexNAc_4_Sia_2_
26	904.3390 (n = 3)	904.3367 (n = 3)	Hex_6_HexNAc_5_Sia_1_Fuc_2_
	1025.7160 (n = 3)	1025.7073 (n = 3)	Hex_7_HexNAc_6_Sia_2_
27	903.9992 (n = 3)	903.9966 (n = 3)	Hex_6_HexNAc_5_Sia_2_
	952.6848 (n = 3)	952.6826 (n = 3)	Hex_6_HexNAc_5_Sia_2_Fuc_1_
28	830.9732 (n = 3)	830.9718 (n = 3)	Hex_5_HexNAc_4_Sia_2_Fuc_1_
29	898.6698 (n = 3)	898.6650 (n = 3)	Hex_5_HexNAc_5_Sia_2_Fuc_1_
	952.6887 (n = 3)	952.6826 (n = 3)	Hex_6_HexNAc_5_Sia_2_Fuc_1_
30	782.2933 (n = 3)	782.2859 (n = 3)	Hex_5_HexNAc_4_Sia_2_
31	830.9772 (n = 3)	830.9718 (n = 3)	Hex_5_HexNAc_4_Sia_2_Fuc_1_
	898.6713 (n = 3)	898.6650 (n = 3)	Hex_5_HexNAc_5_Sia_2_Fuc_1_
32	782.2923 (n = 3)	782.2859 (n = 3)	Hex_5_HexNAc_4_Sia_2_
33	1001.0433 (n = 3)	1001.0284 (n = 3)	Hex_6_HexNAc_5_Sia_3_
34	1049.7169 (n = 3)	1049.7144 (n = 3)	Hex_6_HexNAc_5_Sia_3_Fuc_1_
35	1001.0376 (n = 3)	1001.0284 (n = 3)	Hex_6_HexNAc_5_Sia_3_

Notes: Compositions are given as follows: hexose (Hex), *N*-acetylhexosamine (HexNAc), sialic acid (Sia) and fucose (Fuc).

**Figure 3:**
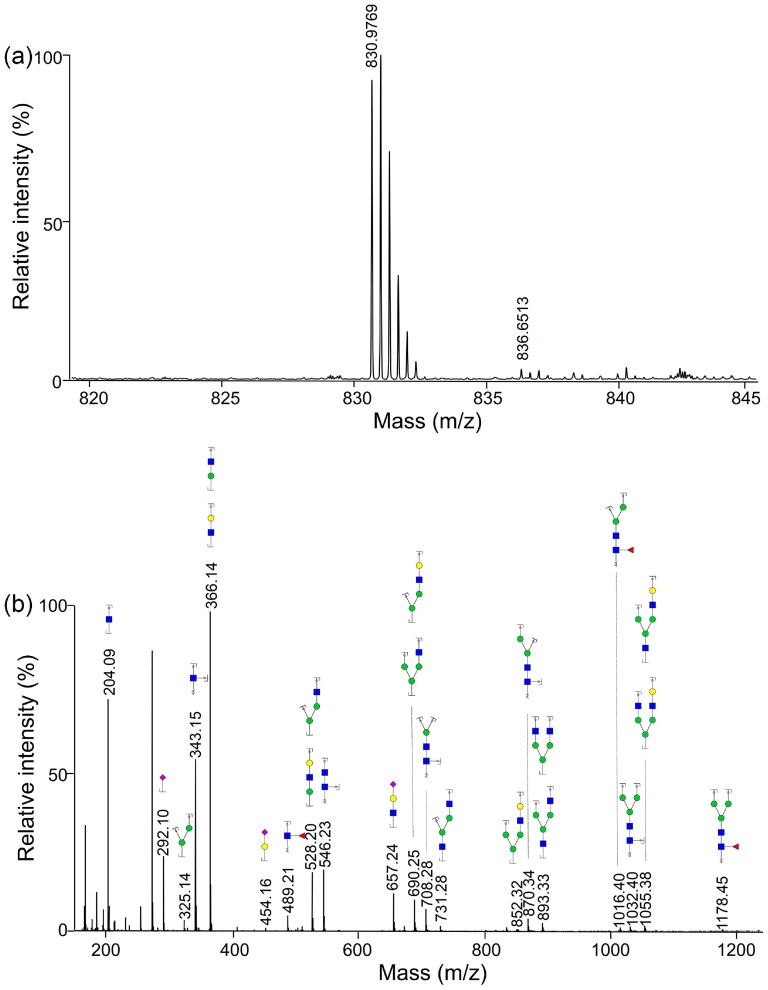
Typical ESI-MS spectrum and MS/MS spectrum from a derivatized glycan. (a) ESI-MS spectrum was obtained for the Hex_5_HexNAc_4_Sia_2_Fuc_1_ with m/z = 830.9769 ([M+3H]^3+^) and m/z = 836.6513 ([M+2H+NH_4_]^3+^). (**b**) Positive ion MS/MS spectrum of Hex_5_HexNAc_4_Sia_2_Fuc_1_ was obtained using an automated data-dependent acquisition mode. Structural schemes are given as follows: blue square, *N*-acetylglucosamine; green circle, mannose; yellow circle, galactose; purple diamond, sialic acid; red triangle, fucose.

Regardless of the isomers, over 40 *N*-glycans were identified by nanoLC-MS and their measured/theoretical masses were listed in Table 2. With an online nano-spray source, most ions were the [M+nH]^n+^ ions without the presence of the [M+(n-m)H+mNa]^n+^ ions. However, a small amount of [M+(n-1)H+NH_4_]^n+^ ions were observed due to the presence of ammonium formate, resulting from the mobile phase of the HPLC analysis. As shown in Figure 3a, two kinds of ions were obtained for the Hex_5_HexNAc_4_Sia_2_Fuc_1_ with m/z = 830.9769 ([M+3H]^3+^) and m/z = 836.6513 ([M+2H+NH4]^3+^). The MS/MS spectra of Hex_5_HexNAc_4_Sia_2_Fuc_1_ was shown in Figure 3b, obtained using the data-dependent acquisition mode and further processed by the GlycoWorkbench 2.1 [35]. The fragment ions with sialic acid residues were limited and the ions with the m/z over 1200 were hardly observed. However, the structures of glycans could be still identified by the MS/MS spectra. For most of the oligosaccharides, the precursor ions with n = 2 and n = 3 could be observed simultaneously, yet the intensities were different, usually being much higher when the m/z was between 700 and 1000. Because of mass-selective sensitivity and ionization efficiency, the glycans with high molecular weight or tri-/tetra sialic acid residues were more difficult to be observed. Only two such oligosaccharides from 10 μL of human serum were identified by nanoLC-MS, although there were some other compositions that might contain tri-/tetra sialic acid residues during HPLC analysis.

### Glycosylation changes in lung cancer

It is well-accepted that aberrant glycosylation patterns exist in cancerous bloodstream. For example, the *N*-glycans from human serum were separated into 17 peaks and five of them were significantly altered in lung cancer [36]. However, these chromatographic peaks all consisted of several glycan compositions that were difficult to be separated. In this paper, a preliminary study about single composition was carried out to evaluate our analytical strategy.

**Figure 4:**
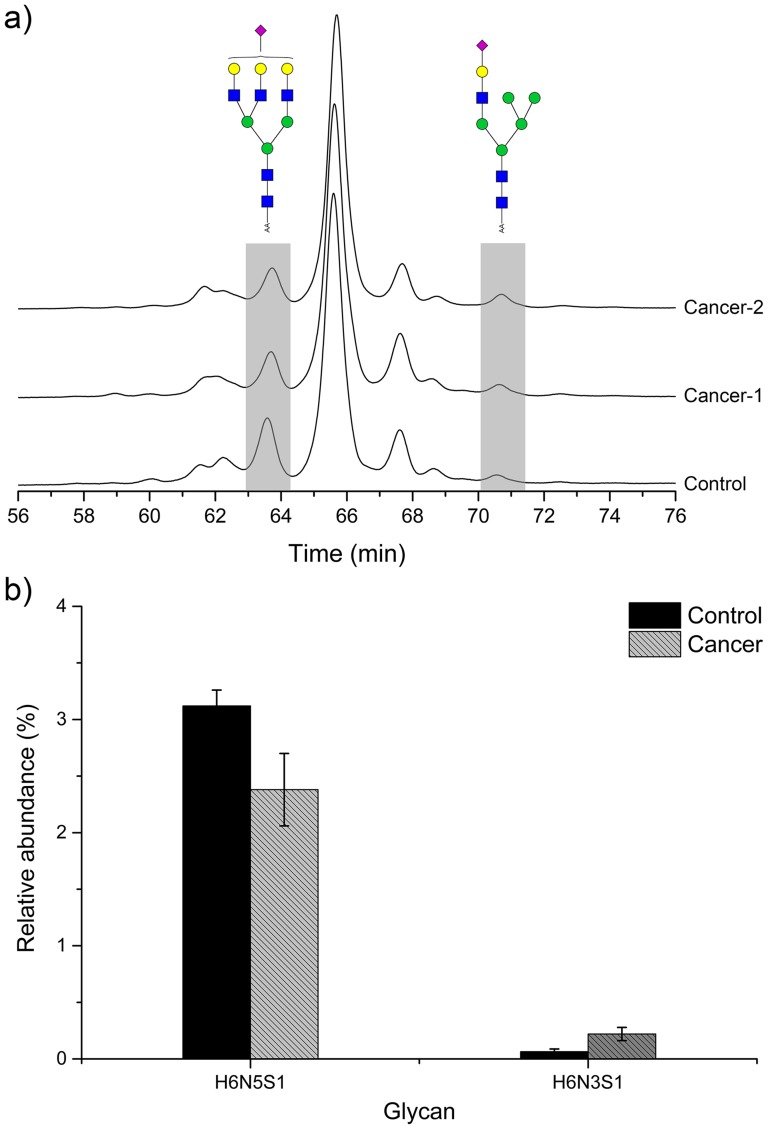
Changes in the glycosylation of the serum samples from the lung cancer patients. (a) Typical HPLC chromatogram showed altered glycosylation from a healthy volunteer and two lung cancer patients. The abundance of Hex_6_HexNAc_5_Sia_1_ was lower in the patients, and the abundance of Hex_6_HexNAc_3_Sia_1_ was higher. (**b**) The bar graph representation of relative average abundances for the Hex_6_HexNAc_5_Sia_1_ and Hex_6_HexNAc_3_Sia_1_ showed significant differences between healthy control groups and lung cancer patients.

The serum samples from 12 individuals, including 6 lung cancer patients and 6 healthy controls, were analyzed by HPLC and the relative abundance of each chromatographic peak was calculated. Figure 4a and 4b showed significant changes from Hex_6_HexNAc_5_Sia_1_ and Hex_6_HexNAc_3_Sia_1_. The relative abundance of Hex_6_HexNAc_5_Sia_1_ was 23.7% lower in the patients than controls, however, the abundance of Hex_6_HexNAc_3_Sia_1_ was 235.9% higher (Figure 4b). The two glycans were preliminarily proved to be associated with lung cancer and might become the potential biomarkers. In addition, Peak 19, consisting of several glycans, was also significantly altered and the compositions were partly same with previous study [36]. It indicated that the human serum *N*-glycome profiling could uncover lung cancer-related changes with some mono-sialylated oligosaccharides and these changes may suggest the involvement of the specific glycoproteins. Overall, due to high sensitivity and resolution, the analytical strategy showed potential applications in the research of serum biomarkers.

## Conclusions

We compared the recoveries and reproducibilities of seven stationary phases for the purification of derivatized oligosaccharides, and further explored the use of MCC as the packing material for SPE cartridges. It is critical to compress the cartridge to approximately 8.5 mm in order to increase sample recovery and repeatability. With the optimized method, the detection limit reached to 0.12 pmol and the recovery was over 70%. Due to the high stability and sensitivity of the MCC cartridge, it showed good performance in the relative quantitative analysis of *N*-glycans from biological samples. Over 40 *N*-glycans from 10 μ μL of human serum were determined by nanoLC-MS and two potential biomarkers were found from the serum samples of lung cancer patients. In summary, we presented an optimized purification method for derivatized glycans, which could be performed with self-packed MCC cartridges. The MCC cartridge has shown potential applications for extremely complex and trace-level samples in glycoproteomic studies.

## Supporting Information

File S1 **This file includes Figure S1 and Table S1.**
(DOC)
