# Supplementary material for: Purification of Derivatized Oligosaccharides by Solid Phase Extraction for Glycomic Analysis
Source: PLoS One. 2014 Apr 4;9(4):e94232. doi: 10.1371/journal.pone.0094232 (PMC3976416; doi:10.1371/journal.pone.0094232)
Supplement: File S1 — This file includes Figure S1 and Table S1. (DOC) [file pone.0094232.s001.doc]

Supporting Information

Purification of derivatized oligosaccharides by solid phase extraction for glycomic analysis

Qiwei Zhang; Henghui Li; Xiaojun Feng*; Bi-Feng Liu and Xin Liu*

*Britton Chance Center for Biomedical Photonics at Wuhan National Laboratory for Optoelectronics–Hubei Bioinformatics & Molecular Imaging Key Laboratory, Systems Biology Theme, Department of Biomedical Engineering, College of Life Science and Technology, Huazhong University of Science and Technology, Wuhan, China*

*Corresponding authors

Address: Department of Biomedical Engineering, College of Life Science and Technology,

Huazhong University of Science and Technology, Wuhan, China

Email: xliu@mail.hust.edu.cn; xfeng@mail.hust.edu.cn

Tel: +86-27-87793180

Fax: +86-27-87792170

**Table S1.** Purification conditions were compared for each type of SPE cartridge.

| **Cartridge** | **Washing solution** | **Elution solution** | **Figure** |
| --- | --- | --- | --- |
| DPA-6S | a-1: 90% ACN (v/v), 5.0 mL | 20% ACN, 1.0 mL | Fig. S1a |
|  | a-2: 90% ACN (v/v), 10.0 mL | 20% ACN, 1.0 mL |  |
|  | a-3: 95% ACN (v/v), 10.0 mL | 20% ACN, 1.0 mL |  |
|  | a-4: 95% ACN (v/v), 5.0 mL | 20% ACN, 1.0 mL |  |
| MCC | b-1: 80% ACN (v/v), 5.0 mL | Water, 1.0 mL | Fig. S1b |
|  | b-2: 80% ACN and 1% FA (v/v), 5.0 mL | Water, 1.0 mL |  |
|  | b-3: 80% ACN and 3% FA (v/v), 5.0 mL | Water, 1.0 mL |  |
|  | b-4: 80% ACN and 5% FA (v/v), 5.0 mL | Water, 1.0 mL |  |
| DSC-CN | c-1: 95% Methanol (v/v), 10.0 mL | Water, 1.0 mL | Fig. S1c |
|  | c-2: 95% Acetone (v/v), 10.0 mL | Water, 1.0 mL |  |
|  | c-3: 80% ACN (v/v), 10.0 mL | Water, 1.0 mL |  |
|  | c-4: 95% ACN (v/v), 10.0 mL | Water, 1.0 mL |  |
| DSC-Si | d-1:Chloroform/Methanol = 1/1 (v/v), 10.0 mL | Water, 1.0 mL | Fig. S1d |
|  | d-2:Acetone/Ethanol = 3/2 (v/v), 15.0 mL | Water, 1.0 mL |  |
|  | d-3:Acetone/Ethanol = 1/1 (v/v), 15.0 mL | Water, 1.0 mL |  |
|  | d-4:Chloroform/Hexane = 1/1 (v/v), 10.0 mL | Water, 1.0 mL |  |
| DSC-NH_2_ | e-1: 80% ACN (v/v), 15.0 mL | Water, 1.0 mL | Fig. S1e |
|  | e-2: 80% ACN (v/v), 15.0 mL | 20% Methanol, 1.0 mL |  |
|  | e-3: 80% ACN (v/v), 15.0 mL | 20% ACN, 1.0 mL |  |
|  | e-4: 80% ACN (v/v), 15.0 mL | 5% FA, 1.0 mL |  |
| DSC-Diol | f-1:Methanol/Water = 9/1 (v/v), 8.0 mL | 20% ACN, 1.0 mL | Fig. S1f |
|  | f-2:Hexane/Acetic acid = 7/3 (v/v), 8.0 mL | 20% ACN, 1.0 mL |  |
|  | f-3:Hexane/Acetic acid = 1/1 (v/v), 8.0 mL | 20% ACN, 1.0 mL |  |
|  | f-4:Hexane/Acetic acid = 3/2 (v/v), 8.0 mL | 20% ACN, 1.0 mL |  |
| ZIC-HILIC | g-1: 90% ACN (v/v), 5.0 mL | Water, 1.0 mL | Fig. S1g |
|  | g-2: 80% ACN (v/v), 5.0 mL | Water, 1.0 mL |  |
|  | g-3: 80% ACN and 1% TFA (v/v), 10.0 mL | Water, 1.0 mL |  |
|  | g-4: 80% ACN and 5% FA (v/v), 10.0 mL | Water, 1.0 mL |  |


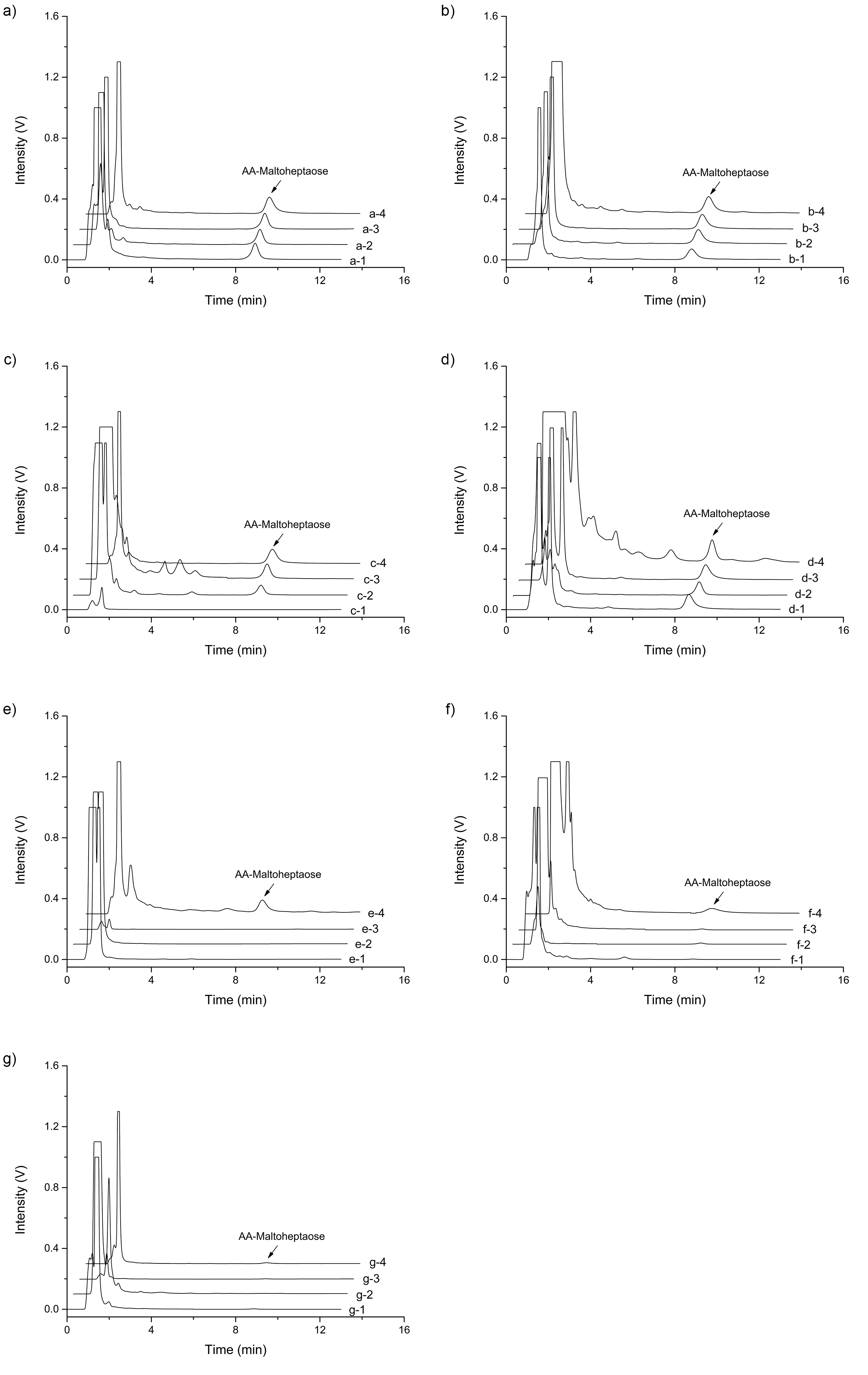


**Figure S1.** The HPLC spectrum corresponding to Table S1. The different purification effect were showed for (**a**) DPA-6S, (**b**) MCC, (**c**) DSC-CN, (**d**) DSC-Si, (**e**) DSC-NH_2_, (**f**) DSC-Diol and (**g**) ZIC-HILIC.
